# Comparative Virulence of Entomopathogenic Nematodes to the Small Hive Beetle (*Aethina tumida* Murray, Coleoptera: Nitidulidae)

**DOI:** 10.2478/jofnem-2025-0011

**Published:** 2025-03-29

**Authors:** George N. Mbata, Kaitlyn Browning, Sanower Warsi, Yinping Li, James D. Ellis, Lambert H. Kanga, David I. Shapiro-Ilan

**Affiliations:** Entomology Research Laboratory, Agricultural Research Station, Fort Valley State University, 1005 State University Drive, Fort Valley, GA 31030, USA; Honeybee Research and Extension Laboratory, Entomology and Nematology Department, University of Florida, Steinmetz Hall, Gainesville, FL 32611-0620, USA; Center for Biological Control, Florida A&M University, 406 Perry-Paige Building, Tallahassee, FL 32307, USA; USDA-ARS, SE Fruit and Tree Nut Research Unit, 21 Dunbar Road, Byron, GA 31008, USA

**Keywords:** small hive beetle, honeybees, beekeepers, entomopathogenic nematodes, persistence of virulence

## Abstract

The small hive beetle (SHB), *Aethina tumida* Murray (Coleoptera: Nitidulidae), has become a ubiquitous, invasive, and highly destructive pest of western honeybee (*Apis mellifera* Linnaeus) hives worldwide. Beekeepers often attempt to control this beetle chemically. Still, ineffective registered control options and rampant off-label chemical use in the beekeeping industry have driven research toward alternative pest management strategies. Entomopathogenic nematodes (EPNs) of the families Heterorhabditidae and Steinernematidae have been established as potential biocontrol agents against soil-dwelling insect pests. However, studies are needed to determine the most appropriate EPN species to control SHB. In this study, an LD_50_ of ~700 infective juveniles (IJs) of EPN per SHB larva was determined through dose-response experiments. This application rate was used to compare the virulence of the following seven species of EPNs against SHB larvae: *Heterorhabditis bacteriophora* (VS), *H. floridensis* (K22), *H. georgiana* (Kesha), *H. indica* (HOM1), *Steinernema carpocapsae* (All), *S. rarum* (17C+E), and *S. riobrave* (355). *Steinernema carpocapsae* (All) and *H. floridensis* (K22) were found to cause 100% larval mortality of SHB at 14 days post-inoculation. Assays for the persistence of virulence of *H. floridensis* (K22) and *S. carpocapsae* in the soil over several weeks from a single application found that both species maintained efficacy, causing 96% mortality of SHB larvae by week 6 post-inoculation. We recommend that *S. carpocapsae* (All) and *H. floridensis* (K22) due to their superior virulence for the control of small hive beetles.

## 1. Introduction

The small hive beetle (SHB), *Aethina tumida* Murray (Coleoptera: Nitidulidae), is a threat to European-derived western honeybee (*Apis mellifera* Linnaeus) populations globally. This beetle is native to sub-Saharan, West, Central, and East Africa (Idrissou et al., 2019), where it is a secondary pest to colonies of African subspecies of *A. mellifera* and preys mainly on weakened colonies (Elzen et al., 2001; Ellis, 2012). Genetic data of SHB from multiple trade routes found that imports of hive products from Ethiopia, South Africa, Tanzania, and the United States are responsible for aiding in the spread of this invasive species (Idrissou et al., 2019).

The SHB has a much more significant impact on colonies of the European subspecies of *A. mellifera* than on colonies of subspecies naturally occurring in Africa (Hayes et al., 2015). The larval stage of this beetle is most destructive to hives, feeding on honey, pollen, and predominantly bee brood (Lundie, 1940). The activity further compromises hive productivity by contaminating and fermenting the remaining honey with SHB fecal deposits (Torto et al., 2007; Valdovinos-Flores et al., 2016). The last instar of the SHB is a non-feeding, wandering stage that exits the hive to pupate in the soil (Hood, 2004). Even healthy colonies of honeybees may collapse due to infestation by the SHB (Ellis, 2012; Hayes et al., 2015). This decline in bee numbers has also led to a decline in genetic diversity among European honeybees (Themudo et al., 2020), leaving hives more susceptible to further losses through inbreeding and the emergence of unfavorable traits. Economically, the value of pollinators’ direct involvement in crop production is estimated at $166 billion (Gallai et al., 2009). However, commercial hives have shown steady declines since the mid-1940s (National Research Council, 2007). Staggeringly, a loss of about 75 to 100 million colonies was reported in the U.S. alone over one winter between 2007 and 2008 (van Engelsdorp et al., 2008). With so much of the world’s food supply reliant on the honeybee, the SHB must be prevented from further decimation of the population of honeybees.

SHBs are developing resistance to broad-spectrum insecticides that have traditionally been used to manage their populations (Kanga et al., 2021; Valdovinos-Flores et al., 2017). Furthermore, the compounds registered for use against SHBs have low efficacy, causing many beekeepers to use off-label chemical control options (Kleckner et al., 2022; Roth et al., 2022). Thus, new pest management tools are needed that are effective in regulating SHB populations and safe for honeybees.

Alternatives to chemical insecticides can be found in biocontrol tools that specifically target key vulnerabilities in the lifecycle of SHBs, such as the wandering larval stage (Sabella et al., 2022). The last instar of SHBs, the wandering larval stage, pupates in the soil outside the hive. This stage provides a “weak link” in the beetle’s lifecycle, where targeted treatments can be most effective. Managing SHB populations at this stage circumvents issues related to the contamination of the hive and reduces the risk of direct contact between honeybees and pesticides.

Entomopathogenic nematodes (EPNs) have been successfully used in the agricultural industry against several soil-dwelling insect pests (Shapiro-Ilan et al., 2020; 2024). EPNs within the roundworm families Heterorhabditidae and Steinernematidae are potent biological control agents (Shapiro-Ilan et al., 2020). They target soil-dwelling insects by entering the hemocoel of the host through the cuticle or any natural orifice during the nematodes’ infective juvenile (IJ) stage (Shapiro-Ilan et al., 2020). Once inside the hemocoel, the EPNs release immune-suppressing agents and enterotoxin symbiotic bacteria that kill the host (Shapiro-Ilan et al., 2020).

Several commercially available EPN species have demonstrated virulence against SHBs, and many of these species have been tested against SHB larvae, with variable degrees of success (Cuthbertson et al., 2012; Ellis et al., 2010; Sanchez et al., 2021). *Steinernema carpocapsae* IJs applied to soil containing SHB wandering larvae have been shown to have continued efficacy for up to 6 weeks after application (Cuthbertson et al., 2012; Cuthbertson et al., 2014). *Steinernema riobrave* and *H. indica* were both shown to have continued efficacy up to 19 weeks following application and generally exhibited superior virulence compared with other EPN species tested (Ellis et al., 2010). In sand assays, *S. carpocapsae* and *S. kraussei* caused 100% mortality of pupating SHBs (Sanchez et al., 2021). In soil assays, *S. riobrave, S. kraussei, H. bacteriophora*, and *H. indica* caused SHB mortality below 35%, with *S. carpocapsae* showing higher efficacy than all others (Sanchez et al., 2021). Based on the variation in results among these prior studies, it is still not clear which EPN species are the most virulent against SHBs.

The rate and frequency of EPN application is of concern for beekeepers as this could raise costs and labor input. Frequent re-application of EPNs in the soil of apiaries may not be needed if there is a supply of suitable soil-dwelling insects on which the EPNs can feed, and better still if EPNs can reproduce on the cadavers of SHBs (Cuthbertson et al., 2012). This could mitigate the costs of using EPNs since re-application would only be needed when there is an outbreak in the host population (Cuthbertson et al., 2012; Ellis et al., 2010). We evaluated seven species of EPNs for virulence to SHBs, and persistence of virulence over time in the soil.

## 2. Materials and Methods

### 2.1. Insect culture

Adult SHBs were obtained from the Honey Bee Research and Extension Laboratory, University of Florida, Gainesville, FL, USA. These were supplemented with wild adult SHBs sourced from infested honeybee hives at Florida A&M University’s Center for Biological Control (Tallahassee, FL, USA). Twenty-five pairs (25 males and 25 females) of adult SHBs were placed on a protein-rich diet comprised of honey, powdered pollen, and bee protein (250, 250, and 500 mL, respectively) in 3.5 L plastic jars and allowed to mate and lay eggs (Neumann et al., 2013). After 14 days, adult SHBs were removed, and the larvae were allowed to develop to the WL (wandering larvae) within the same jars. The WL used in all experiments was collected by placing the diet containing near-matured larvae opposite a hole, allowing migrative WL to be captured in a container placed below the diet container, as described by Neumann et al. (2013). Pupation occurred in chambers filled with sandy-loam soil, and adults emerged after approximately 18 days. The emerged adults were collected using a collection container connected to the pupation container by a funnel. Those that did not migrate into the collection container were collected from the soil surface using an aspirator. All SHB stages were kept in vented 3.8 L plastic containers with lids, in a dark incubator maintained at 25°C, 80% relative humidity. All larvae used in experiments were a maximum of two weeks old.

### 2.2. Soil characteristics

The soil used was composed of sandy loam (Norfolk loamy and sand [Kaolinitic, Thermic Typic Kandiudult]) and was obtained from Fort Valley State University’s research farm located at Fort Valley, GA, USA. The soil had the following characteristics: percentage of sand, silt, and clay = 84:10:6, pH 6.1, organic matter = 2.8% by weight, and moisture = standardized at field capacity (14%) (field capacity is the moisture level a soil will hold against gravity). The soil was autoclaved to eliminate any potentially competing entomopathogens or soil microorganisms. The soil was prepared by placing 5 cm thick layers of soil in aluminum containers, loosely covered with aluminum foil, and heated in an oven at 121 °C for 60 minutes. After heating in an oven, the soil was maintained under laboratory conditions (approximately 25 °C and 55% RH) and aerated for at least two weeks in loosely closed plastic bags to release volatiles. Soil moisture content was determined using the oven method that involved weighing soil samples before and after drying at 60 °C for 24 hours. Percent wet basis moisture content (Mw) was calculated using the formula: $\left(\mathrm{Mw}=\frac{\mathrm{wet}-\mathrm{dry}}{\mathrm{wet}}\times 100\right)$ .

### 2.3. Entomopathogenic nematode cultures

Seven species of entomopathogenic nematodes (EPNs) [*H. bacteriophora* (VS), *H. floridensis* (K22), *H. georgiana* (Kesha), *S. carpocapsae* (All), *S. feltiae* (SN), *S. rarum* (17C+E), and *S. riobrave* (355)] (Table 1) were obtained from the USDA-ARS Fruit and Tree Nut Research Station in Byron, GA. These EPN species were selected based on the results of previous research (Cuthbertson et al., 2012; Ellis et al., 2010; Sanchez et al., 2021; Shapiro-Ilan et al., 2014a) and their commercial availability. EPNs were cultured using commercially procured greater wax moth larvae (*Galleria mellonella* L.) or mealworms (*Tenebrio molitor* L.), following the methods described by Kaya and Stock (1997). Steinernematid IJs were stored at ~ 5°C (Poinar, 1979), while Heterorhabditid IJs were stored at 13°C (Bedding, 1981). All IJs used experimentally were applied within two weeks of the collection date.

**Table 1:** Entomopathogenic nematode strains used in virulence experiments

**Entomopathogenic nematode**	**Strain**	**Abbreviation in manuscript**	**Commercial availability**
*Heterorhabditis bacteriophora*	VS	Hb (VS)	Yes
*H. floridensis*	K22	Hf (K22)	No
*H. georgiana*	Kesha	Hb (Kesha)	No
*H. indica*	HOM1	Hi (HOM1)	Yes
*Steinernema carpocapsae*	All	Sc (All)	Yes
*S. rarum*	17C+E	Sr (17C+E)	No
*S. riobrave*	355	Sr (355)	Yes

### 2.4. Establishing doses of EPNs for experiments

To determine an optimal rate to differentiate EPN virulence in SHB wandering larvae, a three-part process was employed. First, *Hf* (K22), a strain that has been reported to demonstrate a moderate virulence rate compared to other EPNs (Sanchez et al., 2021; Shapiro-Ilan et al., 2014a) was selected for a range-finding experiment to determine the range of doses to test. Then, these doses were used to prepare a dose-response curve (Shapiro et al., 1996), from which the LD50s value was determined. The LD50 was used as a key parameter to investigate the virulence of seven EPN strains.

#### 2.4.1. Range-finding experiment

The range-finding experiment was conducted in 50 mL centrifuge tubes filled with soil, in which the 10 cm depth allowed for natural burrowing activity of both EPNs and SHB wandering larvae (Meikle and Patt, 2011; Meikle and Diaz, 2012; Salame and Glazer, 2015). The autoclaved soil was sun-dried to a moisture content of 0.204% M_w_, as determined by the oven-drying method (Mbata et al., 2022). Fifteen conical centrifuge tubes were filled to the 50 mL mark with the moistened soil, tapping each tube seven times to ensure no air space remained in the soil column. Ten WL were placed on the surface of each soil column and allowed 24 hours to burrow. After this period, the 15 tubes were organized into five treatment sets, with three replications per set. Each set was inoculated with a different concentration of *Hf* (K22) IJs as follows: 0, 25, 200, 500, and 1,000 IJs/larva, applied in 2 mL of aqueous inoculum. Thereafter, tap water was then added to the soil to adjust the volumetric water content to the field capacity (14%). These rates were determined through preliminary assays. The tubes were capped with vented lids and placed inside a plastic bag containing moistened paper towels to maintain high relative humidity (>80% RH) and conserve soil moisture. The tubes were then incubated at 25 °C (Neumann et al., 2013; McMullen, 2014) and checked for larval mortality after 7 days by emptying the tubes onto examination trays and inspecting the soil for live and dead WL.

#### 2.4.2. Dose-response curve

A dose-response curve was established using concentrations determined from the range-finding experiment. As before, 50 mL centrifuge tube soil arenas were prepared, each receiving ten WL added that were allowed 24 hours to burrow. Thereafter, each tube was inoculated with 2 mL of an aqueous solution of *Hf* (K22) at varying concentrations: 0, 50, 100, 200, 400, 800, 1,600, 3,200, and 6,400 IJs/mL, which correspond to 0, 10, 20, 40, 80, 160, 320, 620, and 1,280 IJs/WL, respectively. Five tubes, each containing ten larvae, were inoculated for each concentration. The experiment was set up in a completely randomized design for a total of 45 tubes (450 WL). Control tubes were treated with tap water only (0 IJ/WL). The tubes were capped with vented lids and placed inside a plastic bag with dampened paper towels to maintain high relative humidity. They were incubated at 25°C for 7 days. On the seventh day, the contents of each tube were emptied and checked for SHB larval mortality.

### 2.5. EPN virulence over time

The virulence experiment was conducted using procedures outlined in the previous sections on the dose-response curve (Section 2.4.2) and the evaluation of virulence of EPN species to SHBs (Section 2.4.2), in 50 mL conical centrifuge tubes. The soil was autoclaved, brought to field capacity (14% volumetric water content (VWC)), and transferred to conical centrifuge tubes. The mortality of SHB larvae was evaluated following exposure of the WL to the EPNs for 1-, 2-, 3-, 7-, and 14 days post inoculation (dpi). In this experiment, seven EPN species were used, including all strains listed in Table 1. Fifty mL conical centrifuge tubes were filled to a depth of 45 mL with autoclaved sandy-loamy soil at a moisture content of 14%. Eight WL ≤ 3 weeks old were added randomly to each tube and allowed 24 h to burrow. Thereafter, based on the dose-response experiment, each tube was inoculated with 688 IJs per larva using 2 mL aliquots, setting the final soil VWC to 14%, aligned with the field capacity of sandy loamy soil. Five replicates were prepared for each EPN strain at each time interval. Control tubes received 2 mL of water only. Destructive sampling was carried out at 1-, 2-, 3-, 7-, and 14 dpi for each treatment by pouring out tube contents and searching for both live and infected larvae. The experiment was conducted in four sequential trials over time.

### 2.6. Comparative persistence of nematodevirulence

#### 2.6.1. Persistence of nematode virulence in controlled laboratory conditions

Laboratory assays were conducted to assess the persistence of nematode virulence using an application rate of 100 IJs/cm^2^. Each 50 mL centrifuge tube, having an internal surface area of 5.94 cm^2^, received a total of 600 IJs. *Steinernema carpocapsae* (All), *Hf* (K22), and *Hg* (Kesha) were chosen due to their known virulence towards SHB larvae from previous assays (Shapiro-Ilan et al., 2014a). Two of the strains used, *Sc* (All) and *Hf* (K22), were very virulent to SHBs, while *Hg* (Kesha) was mildly virulent. Tap water was used as a negative control to confirm the virulence of the EPN treatments. Sandy loamy soil with 14% VWC was added to each tube. Thereafter, five WL were selected randomly and placed onto the soil surface and the tubes were then placed in an incubator at 25 °C for 24 h to allow the larvae to burrow into the soil. After 24 hours, the tubes were inoculated with 1 mL of *Sc* (All), *Hf* (K22), *Hg* (Kesha), or tap water in replicates of six. The 1 mL of aqueous inoculum adjusted the final soil VWC to 14%. For the next three consecutive weeks, an additional five SHB WL were introduced weekly to each tube, totaling 20 larvae per tube, or 120 larvae per treatment. The tubes were monitored weekly for six consecutive weeks post-inoculation to check for emerging adults. Any emerged adults were counted and removed from the tubes. Wandering larvae that failed to develop into adults during these checks were considered dead. A final destructive search of the soil tubes was conducted on the seventh-week post-inoculation to confirm the count of deceased larvae. This experiment was repeated five times.

#### 2.6.2. Persistence of nematode virulence in the greenhouse

As a follow-up experiment to the one outlined in 2.6.1, a greenhouse experiment was conducted to determine the persistence of virulence of EPNs and to test the nematode spray method planned for subsequent field experiments. Cylindrical plastic containers (21 cm height, 16.5 cm top diameter, 14 cm bottom diameter, and 3.8 L of total volume) were filled with sandy-loamy soil to a depth of 11 cm. The containers were filled with soil taken directly from the field. They had a mean moisture content of 13.85 (± 1.3) %, determined using a General DSMM500 soil moisture meter with probe (Worthington Industries, Greenville, TN, USA).

The containers with soil were taken to the laboratory, where 30 randomly selected WL were added to each container and allowed 24 h to burrow. To simulate a possible field application method, a SOLO 430-2G sprayer (SOLO incorporated, Newport News, VA, USA) equipped with a TeeJet AI-11003 nozzle (Teejet Technologies, Glendale Heights, IL, USA) was used to apply aqueous IJ suspensions (Shapiro-Ilan et al., 2010) to the soil. This nozzle was chosen for its ability to produce coarse droplets (Moreira et al., 2013). During preliminary trials, the use of this nozzle resulted in an EPN survival rate of 91.34% after spraying. It provided a precise application rate of 0.63 mL/sec at 310 kPa, which was an acceptable low rate of spray that ensured accuracy (± 1.12 mL). Infective juveniles of *Hf* (K22) and *Sc* (All) were applied at a rate of 100 IJs/cm^2^ (area of 176.71cm^2^ at the soil surface) and a volume of 100 mL per container for a total of 17,760 IJs/container.

Five replications of each treatment [*Sc* (All), *Hf* (K22), and a water control] were completed for a total of fifteen containers and 150 initial WL per treatment, with a total of 450 WL across all treatments. Following the application of IJs, the containers were placed in the greenhouse with 200 mL of tap water added biweekly to maintain moist soil and prevent visible soil dryness. Seven days post IJ inoculation, baited traps were deployed in each container to attract and capture emerging adult SHBs. Traps consisted of 29.5 mL plastic cups filled with apple cider vinegar as an attractant and mineral oil to prevent escape (Hood and Miller, 2003). Punctured lids were applied to allow adult SHB entrance and prevent spillage of the contents. Following this, lids were placed on each soil container. The surfaces of the lids were replaced with polyethylene mesh (Agfabric Insect Barrier Netting, Vista, CA, USA). Emerged adults were collected from each container every seven days, and 30 new SHB WL were added every 14 days. The first round of WL was applied on 18 Aug. 2023 and the last round on 15 Sept. 2023, for 90 larvae added to each container over this period. The final collection of adults was completed on 22 Sept. 2023.

### 2.7. Data analysis

**Figure 1:**
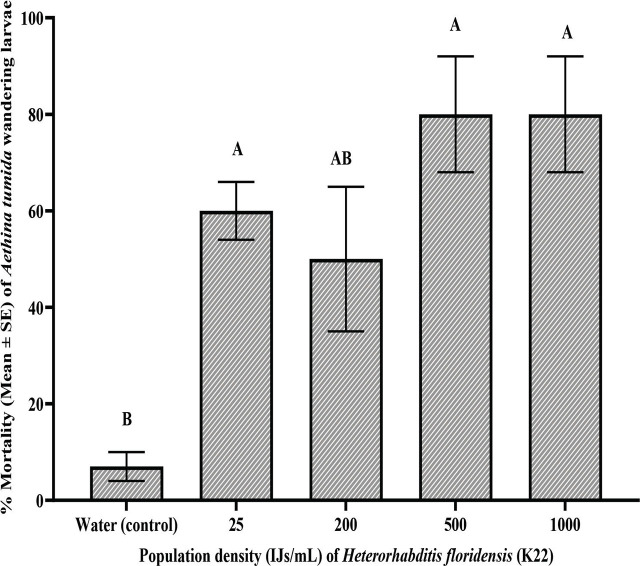
Percent seven-day mortality (Mean ± s.e.) of *Aethina tumida* wandering larvae after treatment with four population densities of *Heterorhabditis floridensis* (K22). The data were graphed with one-way ANOVA using Tukey’s Kramer multiple comparison test. Different letters above the bars indicate significant differences (*P* < 0.05).

Data was analyzed using SAS ver. 9.4 (SAS Institute, Cary, NC, USA) for most statistical tests. The dose-response curve and other graphs were generated with GraphPad Prism ver. 10.1.1 (GraphPad Software, Boston, Massachusetts, USA). In preparing the dose-response curve, doses were converted to log10, and percentage mortality was plotted to estimate LD50 values. Data met ANOVA assumptions of normal distribution (Shapiro-Wilk, Cramer-von Mises, Anderson-Darling, Kolmogorov tests) and homogeneity of variance (Bartlett’s, Brown-Forsythe, Levene’s tests). ANOVA was subsequently applied, and when results were significant, multiple means comparisons were conducted using Tukey-Kramer’s post-hoc test (*P* ≤ 0.05). One-way ANOVA was used to compare SHB larval mortality across different EPN species/strains in range-finding experiments (Fig. 1), virulence over time (Table 2), and nematode virulence in controlled laboratory conditions (Fig. 3 & 4), while two-way ANOVA was applied to assess the combined effects of time (weeks) and EPN species/strains on mortality in the greenhouse persistence experiment (Table 3).

**Table 2:** Mortality (%) of *Aethina tumida* wandering larvae after treatment with one of seven entomopathogenic nematodes (EPN) species after one of five inoculation days (1-, 2-, 3-, 7-, or 14-days post inoculation).

**Days post inoculation**	**Mean ± S.E.**							

	**Water (control)**	***Heterorhabditis bacteriophora* (VS)**	***Heterorhabditis floridensis* (K22)**	***Heterorhabditis georgiana* (Kesha)**	***Heterorhabditis indica* (HOM1)**	***Steinernema carpocapsae* (All)**	***Steinernema rarum* (17c+e)**	***Steinernema riobrave* (355)**
1	0	0	3 ± 3	0	0	0	0	0
2	0	3 ± 3	10 ± 5	8 ± 4	5 ± 5	5 ± 3	0	3 ± 3
3	0c	13 ± 4abc	10 ± 5abc	8 ± 4abc	5 ± 3bc	25 ± 7ab	30 ± 8a	5 ± 3bc
7	3 ± 3d	53 ± 5bc	88 ± 4a	44 ± 8c	53 ± 12bc	100a	80 ± 8ab	88 ± 6a
14	10 ± 3d	70 ± 5bc	100 ± 4a	78 ± 6ab	45 ± 10c	100a	78 ± 11ab	93 ± 5ab

Means followed by different lowercase letters in each row are significantly different (*P*< 0.05) among EPN strains within each incubation day (dpi).

**Figure 2:**
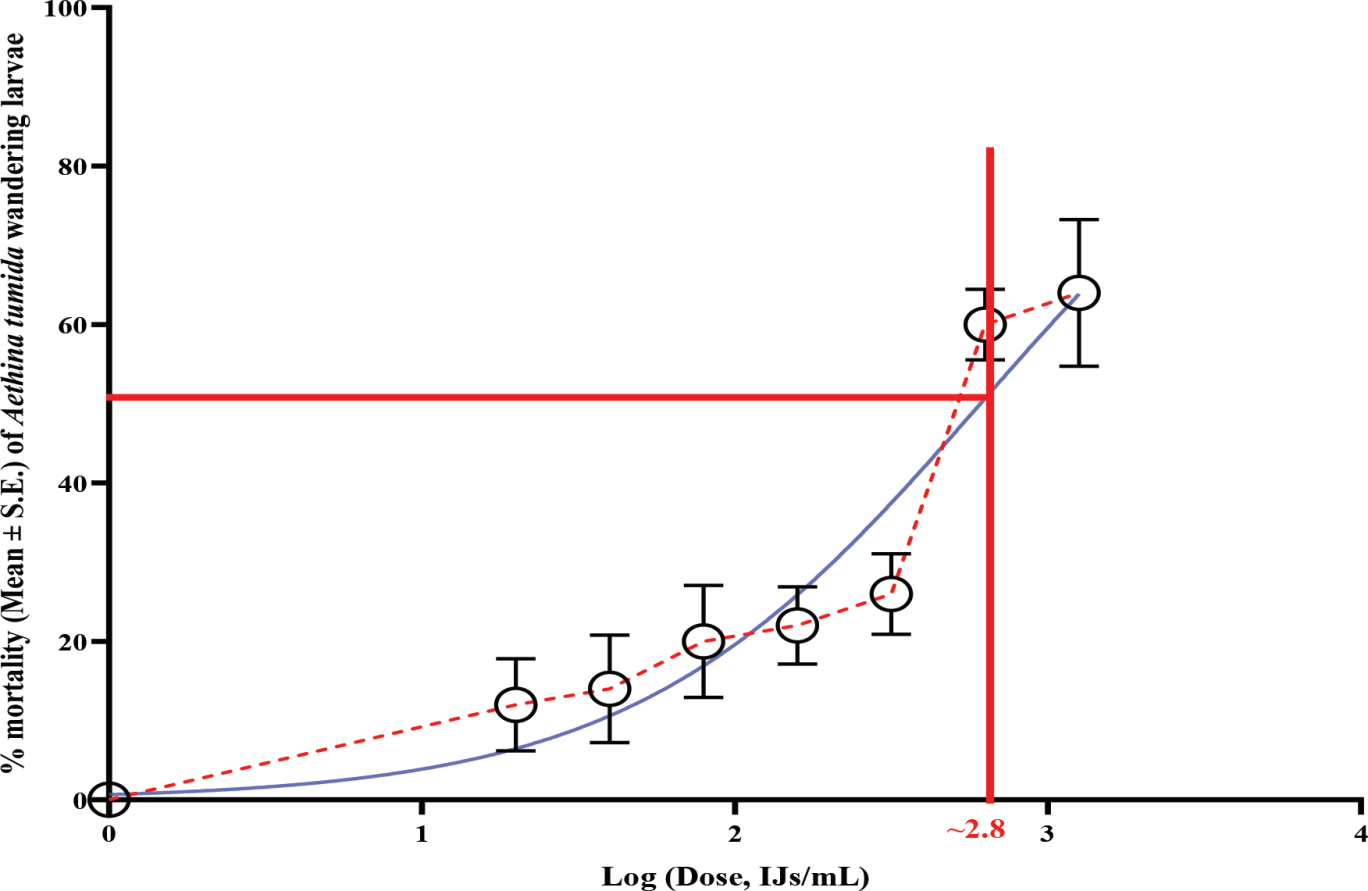
Dose-response curve showing the mortality of *Aethina tumida* wandering larvae given increasing doses of *Heterorhabditis floridensis* (K22). The dashed red curve represents actual observations. Solid red lines show the intersection of the log dose and 50% mortality to find the LD_50_, represented by the black star. The model fit was evaluated with R^2^ = 0.70.

**Figure 3:**
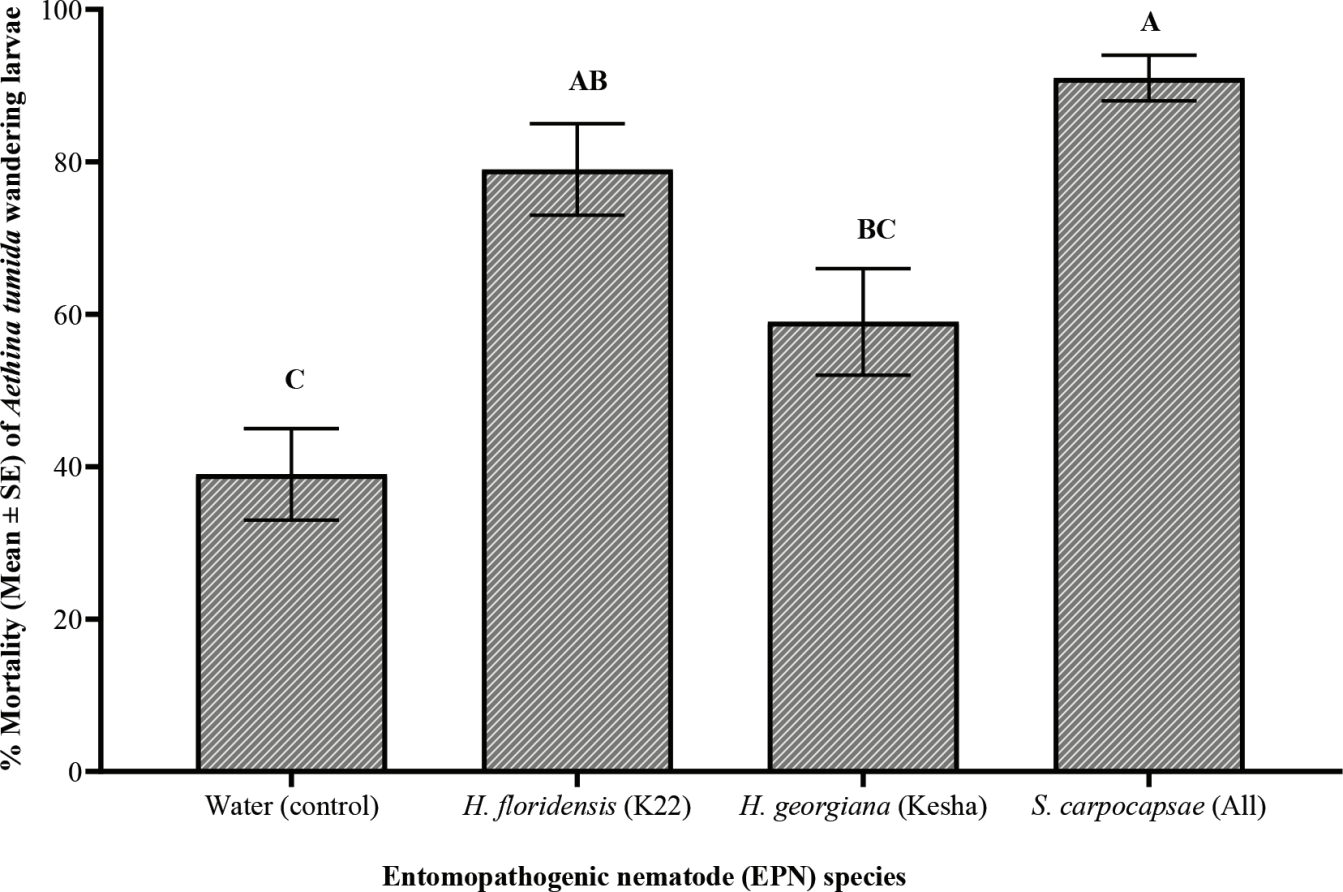
Percent mortality (Mean ±SE) of *Aethina tumida* wandering larvae after treatment with one of three entomopathogenic nematodes (EPN) species: *Steinernema carpocapsae* (All), *Heterorhabditis floridensis* (K22), and *H. georgiana* (Kesha) after different inoculation weeks (3, 4, 5, or 6 weeks post-inoculation) in the laboratory persistence experiment. The data were graphed with one-way ANOVA using Tukey’s Kramer multiple mean comparison test. Different letters above the bars indicate significant differences (*P* < 0.05).

**Figure 4:**
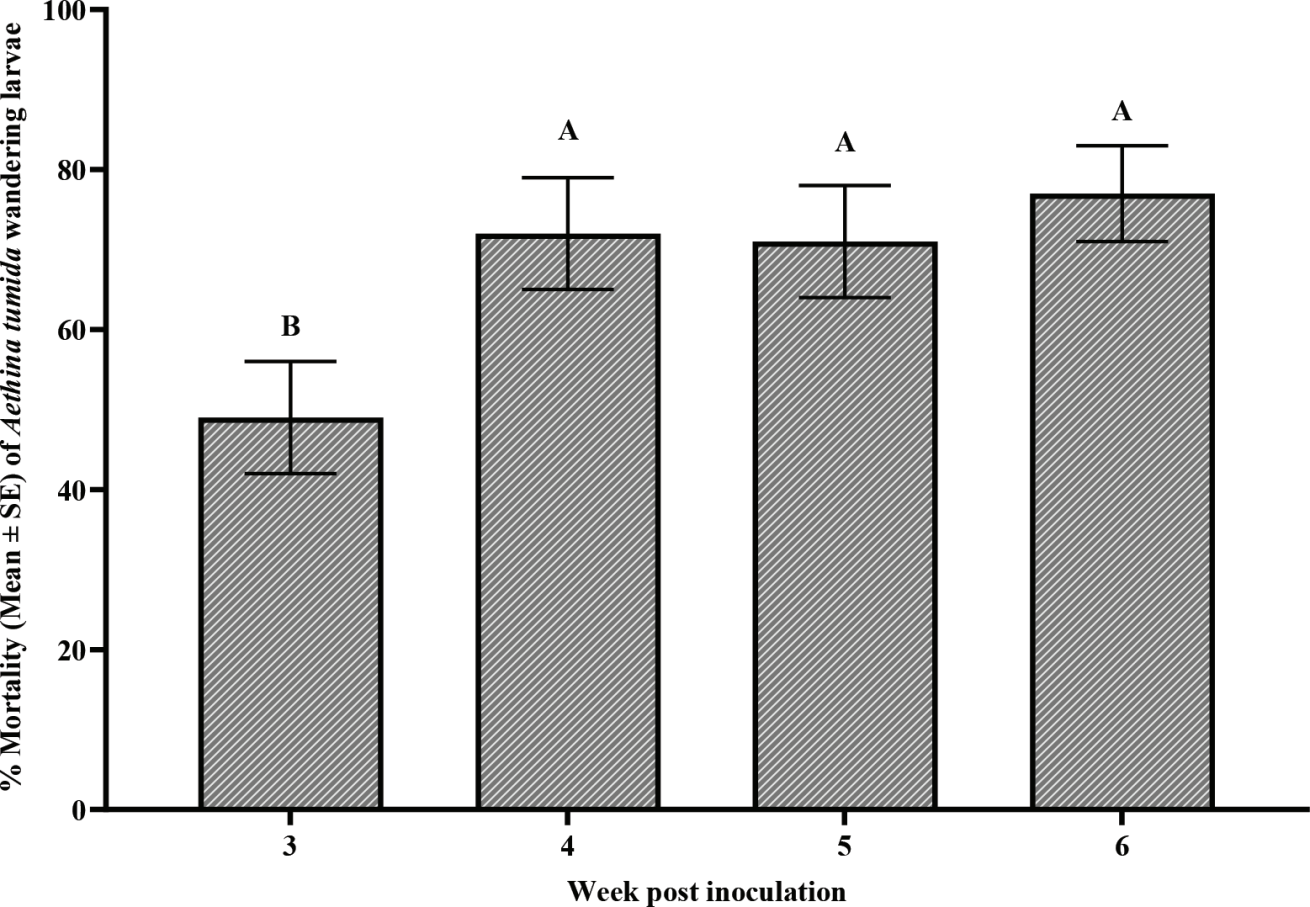
Percentage mortality (Mean ±SE) of *Aethina tumida* wandering larvae following inoculation with entomopathogenic nematodes infective juveniles after four different inoculation weeks (3-, 4-, 5-, or 6-weeks post inoculation) in the laboratory persistence experiment. The data were pooled utilizing three nematode strains. Different letters above bars indicate significant differences (*P* < 0.05, Tukey’s Kramer multiple mean comparison test).

**Table 3:** Mean percent mortality (± s.e.) of *Aethina tumida* wandering larvae after treatments with two entomopathogenic nematodes (EPN) species after six incubation weeks (1-, 2-, 3-, 4-, 5-, and 6 weeks post-inoculation) in the greenhouse persistence experiment.

**Week post inoculation**	**Water (control)**	***Heterorhabditis floridensis* (K22)**	***Steinernema carpocapsae* (All)**
1	28 ± 10.1aB	70.6 ± 6.27bA	86 ± 6.86abA
2	28 ± 10.1aB	84 ± 5.31abA	85.33 ± 4.42abA
3	28 ± 10.1aB	100 ± 0.00aA	98.66 ± 0.81aA
4	44 ± 23.1aA	72.66 ± 3.71bA	69.33 ± 3.85bcA
5	44 ± 13.2aA	68 ± 5.22bA	56.66 ± 5.27cA
6	44 ± 13.2aB	93.33 ± 1.05aA	94.66 ± 2.70aA

Means followed by different lowercase letters in each column are significantly different (*P* < 0.05) among EPN strains within each incubation week. Means followed by different uppercase letters in each row significantly differ (*P* < 0.05, Tukey’s test) among incubation weeks within each EPN strain.

## 3. Results

### 3.1. Range finding and dose-response

#### 3.1.1. Range-finding experiment and dose-response curve

Nematode concentration significantly affected the percentage mortality of SHB larvae (F = 8.35, df = 4, 10, *P* = 0.0032). The mortality of SHB larvae increased significantly compared to the control, but none of the nematode concentrations were significantly different from one another (Fig. 1). The control group, treated with water, showed a minimal mortality of 7.0 ± 3.0%, significantly lower than that of the nematode-treated groups. A concentration of 25 IJs/mL of *Hf* (K22) resulted in a mortality of 60.0 ± 6.0%, which was notably higher than mortality in the control group but not significantly different from that of the 200 IJs/mL treatment, which achieved a 50.0% ± 15.0% mortality. The higher nematode concentrations, 500 and 1000 IJs/mL, resulted in the highest mortality of 80.0 ± 12.0% from the lower concentrations (25 and 200 IJs/mL).

The dose-response curve (Fig. 2) showed an increase in mortality corresponding to increasing doses of *Hf* (K22) IJs until reaching a threshold at approximately 700 IJ/WL, which corresponded to an LD_50_ = 700 IJ/WL.

### 3.2. Virulence over time

There was a significant effect of nematode species/strain on the mortality of SHB (F = 41.91, df = 7, 152, *P* < 0.0001; Table 2). At 1 and 2 dpi, no significant differences existed in the virulence of EPN species/strains. Mortality in SHB larvae remained low across all treatments (Table 2). At 3 dpi, moderate mortality was observed, with *Sr* (17C+E) causing 30% mortality, though it was not significantly different from *Sc* (All), *Hf* (K22), *Hb* (VS), and *Hg* (Kesha). The most notable differences in virulence among EPN species were observed at 7 dpi, where *Sc* (All) caused 100% mortality. This was followed closely by *Sr* (355) and *Hf* (K22), both causing 88% mortality, and *Sr* (17C+E), which caused 80% mortality. However, these four species/strains were not significantly different from each other at this time point. At 14 dpi, *Sc* (All) and *Hf* (K22) remained the most virulent species, consistently causing 100% mortality, while *Sr* (355) and *Sr* (17C+E) caused 93% and 78% mortality, respectively. In contrast, *Hi* (HOM1) showed the lowest efficacy, causing approximately 45% mortality by the end of the observation period. In water (control), peak mortality reached 10% at 14 dpi.

### 3.3. Comparative persistence of nematode virulence

#### 3.3.1. Persistence of nematode virulence in controlled laboratory conditions

The persistence of the EPN species virulence over time in laboratory conditions was significantly different (F = 8.41, df = 3, 20, *P* = 0.0008; Fig. 3). The control treatment resulted in minimal larval mortality compared to mortality among larvae in the EPN treatments. *Sc* (All) numerically exhibited the highest mortality, maintaining close to 100% efficacy throughout the study period. However, the treatment was not significantly different from *Hf* (K22) (Fig. 3). Hg (Kesha) exhibited moderate efficacy, causing significantly lower mortality of SHBs than mortality for SHB larvae treated with Sc (All). Over time, nematode efficacy persisted consistently (F = 6.70; df = 3, 60; *P* = 0.0006; Fig. 4). Week 3 mortality was significantly lower than subsequent weeks. However, from weeks 4 to 6, SHB larvae mortality remained high and stable, with no significant differences among these weeks. However, the interaction between EPN species/strain and incubation week had no significant effect on SHB larval mortality (F = 1.16; df = 9, 60; *P* = 0.336).

#### 3.3.2. Persistence nematode virulence in the greenhouse

The virulence of the EPNs measured by the mortality of SHB larvae introduced weekly over a six-week period to soil inoculated with two entomopathogenic nematodes, *Hf* (K22) and *Sc* (All), is shown in Table 3. Inoculation of SHBs with EPNs had significant effect on the mortality of SHB larvae across different EPN species/strains (F = 38.50, df = 2, 72, *P* < 0.0001), periods of introduction of SHB larvae following inoculation (F = 2.58, df = 5, 72, *P =* 0.05), and the interaction between EPN species and inoculation time (F = 4.0, df = 10, 72, *P* = 0.0003). In weeks 1 to 3, *Hf* (K22) and *Sc* (All) caused significantly higher mortality than the control, with no differences between the two species. Mortality declined in weeks 4 and 5, and neither EPN species significantly differed from the control. In week 6, both EPNs regained high efficacy, with mortality significantly higher than the control.

The mortality of SHB larvae was significantly higher for the EPN treatments than that of the control. Inoculation of SHBs with EPNs had a significant effect on the mortality of SHB larvae over six weeks (F = 38.50, df = 2, 72, *P* < 0.0001). The virulence of the EPNs exhibited slight variation across the weeks, while the mortality of SHB larvae exposed to IJs of a similar strain did not vary significantly. However, high mortality (> 90%) of SHB larvae exposed to *Hf* (K22) or *Sc* (All) inoculated soil in the sixth week was recorded (F = 2.58, df = 5, 72, *P =* 0.05). The mortality of SHB larvae was highest in the third and sixth weeks of observations. The interaction between the EPN strain and periods of the introduction of SHB larvae following inoculation was significant (F = 4.0, df = 10, 72, *P* = 0.0003). Indeed, in the later weeks of the experiment, IJs of *Hf* (K22) and *Sc* (All) were found to have aggregated near the vents of the caps of their respective tubes, which reflected high populations of IJs in the soil that resulted in some migrating to the caps of the vials.

## 4. Discussion

We investigated the efficacy and persistence of different EPN species in managing *SHB* larvae, focusing on mortality rates across different doses, time points, and environmental conditions. Our range-finding experiment results showed that increasing the *Hf* (K22) IJs concentration resulted in higher SHB larval mortality, reaching a threshold where further increases in nematode concentrations did not enhance efficacy. The close alignment of the fitted curve with the empirical data points confirmed the adequacy of our model in capturing the dose-response relationship in both the range-finding and dose-response experiments, larval mortality plateaued at or below 80%, even at high application rates of 1000 and 1,280 IJs/WL. These concentrations exceeded the standard EPN application rate for highly susceptible hosts like wax moth larvae (Alonso et al., 2018). This result aligns with the findings of Shapiro-Ilan et al. (2014a), who also reported moderate *Hf* (K22) efficacy towards SHB larvae, reinforcing the need to balance dose with pest-specific efficacy.

Compared to the control (water), all EPN strains tested were markedly more effective in parasitizing SHB larvae. *Steinernema carpocapsae* (All) and (K22) exhibited the highest virulence, achieving 100% mortality in beetle larvae at the end of the observation period. In contrast, *Hb* (VS), *Hg* (Kesha), and *Hi* (HOM1) showed the lowest virulence. Previous studies have demonstrated the high infectivity of *Sc* (All) to SHB larvae (Ellis et al., 2010; Sanchez et al., 2021). Our study supports these findings, revealing comparable levels of virulence between *Sc* (All) and *Hf* (K22) against the SHB, consistent with earlier observations by Shapiro-Ilan et al. (2014a).

A range of features may impact and facilitate the infectivity of nematodes to insects, including foraging behavior and size of nematode IJs. Host insect mobility and the foraging behavior of an EPN may also affect the susceptibility of an insect to an EPN (Lewis et al., 1992; Griffin, 2012). *Steinernema carpocapsae* (All) is an ambusher, targeting mobile hosts, thus making it highly effective against wandering SHB larvae, which are actively searching for pupation sites (Lewis et al., 1992; Griffin, 2012). Since SHB larvae naturally move through the soil, they are more likely to encounter *Sc* (All), leading to high infection rates. In contrast, *Hf* (K22) demonstrates cruiser behavior, allowing it to cover more ground and locate fewer mobile hosts that have begun the pupation process (Rezaei et al., 2015). By actively searching for hosts, *Hf* (K22) ensures successful infection, even when larvae are no longer wandering.

Although several of the tested nematode strains in this study caused significant mortality, *Sc* (All) was the only species to reach 100% mortality by 7 dpi. This quick and effective infection, coupled with its high tolerance to desiccation and UV radiation, makes *Sc* (All) an ideal candidate for application to soil surfaces in apiaries, where environmental stress can limit other biocontrol agents (Shapiro-Ilan et al., 2014b, 2015). While *Hf* (K22) has lower desiccation tolerance, it has a broad temperature range, including a high level of heat tolerance. It may be suitable for the treatment of apiaries under diverse conditions (Shapiro-Ilan et al., 2014a).

Our persistence experiments provide valuable insights into the long-term effectiveness of *Sc* (All) and *Hf* (K22) against SHB larvae. Both species maintained high virulence throughout the six-week study period, with a general trend of sustained SHB larval mortality. However, a temporary decline in mortality was observed in weeks 3, 4, and 5, likely due to the natural die-off of the initial EPN population. The subsequent resurgence in mortality (80–90%) towards the end of the experiment was possibly due to EPN reproduction inside infected SHB larvae, leading to a new wave of infective juveniles. Griffin (2015) also indicated that EPN infectivity can increase after extended storage (4–6 weeks), which may have also contributed to the observed increase in mortality during the later stages of the experiment.

Importantly, both *Sc* (All) and *Hf* (K22) maintained their effectiveness throughout the study, indicating that a single application of IJs could provide reliable control over several weeks. These findings align with studies by Ellis et al. (2010), which demonstrated that certain EPN strains like *Sr* (355) and *Hi* (HOM1) can persist in the soil for up to 19 weeks post-application. Interestingly, in both laboratory and greenhouse conditions, *Hf* (K22) exhibited comparable efficacy to *Sc* (All), with mortality of SHB larvae remaining consistently high across weeks. This sustained performance may be explained by the reproduction of *Hf* (K22) in the soil. In the later stages of our laboratory experiments, large aggregations of IJs from both nematodes were observed near the vent caps of their vials, suggesting high IJ population densities and continued reproduction (White, 1927). These results highlight the ecological advantages of *Sc* (All) as a biocontrol agent. This species has been documented to perform well in diverse soil types, even under low-moisture conditions, albeit at higher concentrations (~1,941 IJs per mL) (Cuthbertson et al., 2012). The tolerance of *Sc* (All) to desiccation further enhances its suitability for field applications on soil surfaces in apiaries (Shapiro-Ilan et al., 2014b).

The persistence of *Sc* (All) and *Hf* (K22) demonstrates their value in integrated pest management programs targeting SHB. The continuous mortality observed with the reintroduction of fresh SHB larvae emphasizes that both *Sc* (All) and *Hf* (K22) can offer sustained suppression. Further research is needed to determine the optimal frequency of application and assess how environmental factors, such as UV exposure, impact their efficacy in field settings (Shapiro-Ilan & Goolsby, 2021). These findings underscore the potential of these entomopathogenic nematodes to play a crucial role in sustainable pest control strategies within the beekeeping community (Hoffmann et al., 2008). Efficacy and persistence of the EPN strains that indicated high potential in our study should be explored further in field trials.
